# Species‐specific clinical characteristics of human coronavirus infection among otherwise healthy adolescents and adults

**DOI:** 10.1111/irv.12538

**Published:** 2018-02-02

**Authors:** Monique Bouvier, Wei‐Ju Chen, John C. Arnold, Mary P. Fairchok, Patrick J. Danaher, Tahaniyat Lalani, Leslie Malone, Deepika Mor, Michelande Ridoré, Timothy H. Burgess, Eugene V. Millar

**Affiliations:** ^1^ Betty Irene Moore School of Nursing University of California Davis Sacramento CA USA; ^2^ Department of Preventive Medicine and Biostatistics Infectious Disease Clinical Research Program Uniformed Services University of the Health Sciences Bethesda MD USA; ^3^ Henry M. Jackson Foundation for the Advancement of Military Medicine Bethesda MD USA; ^4^ Naval Medical Center San Diego CA USA; ^5^ Madigan Army Medical Center Tacoma WA USA; ^6^ San Antonio Military Medical Center San Antonio TX USA; ^7^ Diatherix Laboratories, LLC Huntsville AL USA; ^8^ Children's National Medical Center Washington DC USA

**Keywords:** clinical characteristics, coronavirus, influenza‐like illness

## Abstract

Human coronavirus (HCoV) is a known cause of influenza‐like illness (ILI). In a multisite, observational, longitudinal study of ILI among otherwise healthy adolescents and adults, 12% of subjects were PCR‐positive for HCoV. The distribution of species was as follows: HCoV‐OC43 (34%), HCoV‐229E (28%), HCoV‐NL63 (22%), and HCoV‐HKU1 (16%). We did not observe species‐specific differences in the clinical characteristics of HCoV infection, with the exception of HCoV‐HKU1, for which the severity of gastrointestinal symptoms trended higher on the fourth day of illness.

## 1 INTRODUCTION

Clinical manifestations of human coronavirus (HCoV) infection range from a mild, self‐limiting illness of the upper respiratory tract to an acute respiratory distress syndrome with a high mortality rate. Highly virulent species of HCoV were responsible for outbreaks of severe acute respiratory syndrome (SARS) and Middle East respiratory syndrome (MERS); case‐fatality rates ranged from 14% to 45%.^1^, ^2^, ^3^ By contrast, other HCoV species (HCoV‐HKU1, HCoV‐OC43, HCoV‐NL63, and HCoV‐229E) are much more prevalent, much less severe, and common causes of influenza‐like illness (ILI).^4^, ^5^, ^6^, ^7^, ^8^, ^9^, ^10^, ^11^ Five previous studies have described the species‐specific clinical characteristics of HCoV infection among adults.^6^, ^7^, ^10^, ^11^, ^12^ In two of these studies, a significant proportion of the study population had underlying medical conditions.^6^, ^7^ Herein, we describe, among a cohort of otherwise healthy adolescents and adults with influenza‐like illness (ILI), the species‐specific prevalence and severity of symptoms associated with HCoV infection.

## 2 METHODS

The Acute Respiratory Infection Consortium (ARIC) is a multisite, multidisciplinary clinical research network for the study of ILI in US Department of Defense (DoD) healthcare facilities. Established in 2009, the ARIC Natural History Study is an observational, longitudinal cohort study of the etiology, epidemiology, and clinical characteristics of ILI among otherwise healthy DoD members and beneficiaries.^13^ Patients 0‐65 years of age and presenting for care <72 hours after onset of ILI symptoms were recruited for study participation. ILI was defined as a temperature ≥100.4°F and sore throat or one of the following respiratory symptoms: cough, sputum production, shortness of breath, or chest pain. Both inpatient and outpatient subjects were eligible to participate. Patients with underlying medical conditions (eg, diabetes, chronic obstructive pulmonary disease, severe asthma), women with a high‐risk or complicated pregnancy, and patients with a poorly controlled psychiatric disorder were excluded.

A nasopharyngeal (NP) swab (Nylon‐flocked, Copan Diagnostics, Corona, CA) was collected at baseline (ie, enrollment) for the detection of respiratory pathogens. Swabs were placed immediately into viral transport media, frozen at −80°F, and shipped on dry ice to the Naval Health Research Center (San Diego, CA). All specimens were tested for influenza by the CDC human influenza virus real‐time reverse transcription polymerase chain reaction (rtRT‐PCR). An aliquot was then tested by TEM‐PCR^™^ (Diatherix Laboratories, LLC; Huntsville, AL), a multiplex PCR‐based assay for the detection of viral and bacterial respiratory pathogens. Viral targets on TEM‐PCR^™^ include influenza A, influenza B, respiratory syncytial virus (RSV) types A and B, rhinovirus/enterovirus, parainfluenza virus types 1‐4, human metapneumovirus (hMPV), adenovirus types B and E, and coronavirus species 229E, NL63, OC43, and HKU1.

Information on patient demographics and presence/severity of symptoms at the time of enrollment was collected by in‐person interview. Participants were then instructed on the use of a daily diary to record the presence/severity of symptoms for 7 days following initial symptom onset. Symptom severity was rated on an ordinal scale from 0 (none) to 3 (severe). Symptom severity scores were quantified using the following five measures: (i) individual symptom score for 20 symptoms, (ii) the upper respiratory symptom score, calculated as the sum of severity scores for earache, runny nose, sore throat, and sneezing, (iii) the lower respiratory symptom score, calculated as the sum of severity scores for cough, difficulty breathing, hoarseness, and chest discomfort, (iv) the gastrointestinal symptom score, calculated as the sum of severity scores for diarrhea, vomiting, anorexia, nausea, and abdominal pain, and (v) the composite systemic symptom score, calculated as the sum of severity scores for chills, muscle ache, headache, and fatigue.

Differences in patient demographics, study site, and ILI risk factors by HCoV species were compared using descriptive statistics (eg, chi‐square or Fisher's exact tests). Composite symptom scores and 95% confidence intervals (CI) were calculated to evaluate between species differences in clinical characteristics. Statistical comparisons were performed using Kruskal‐Wallis tests. Because this was an exploratory analysis of species‐specific differences in symptom severity, corrections for multiple comparisons were not made. Analyses were performed using SPSS (version 22.0; IBM Corporation, Armonk, NY).

The study was approved by the Infectious Disease Institutional Review Board of the Uniformed Services University of the Health Sciences (IDCRP‐045).

## 3 RESULTS

Between 2009 and 2014, a total of 902 participants were enrolled in the ARIC NHS and had a baseline nasopharyngeal specimen evaluated by TEM‐PCR. Of these, 111 (12%) were positive for HCoV. Cases occurred from September through June with the highest number (34%) of cases occurring in February. Fourteen (12.6%) cases were positive for another viral respiratory pathogen, including human rhinovirus (57.1%), adenovirus (21.4%), influenza A (14.3%), and influenza B (7.1%). Fifteen (13.5%) cases were <13 years of age. These two groups of subjects were excluded from analysis of symptom severity. Of the remaining 82 cases, the mean (range) age was 28.4 (13.2‐49.9) years. Forty‐nine (60%) were white, 42 (51%) were female, and 69 (84%) were active duty military members (Table 1). One subject was hospitalized (duration: 1 day).

**Table 1:** Demographic and epidemiologic characteristics of 82 adolescents and adults with human coronavirus infection by species, 2009‐2014

	229E n = 23 N (%)	NL63 n = 18 N (%)	OC43 n = 28 N (%)	HKU1 n = 13 N (%)	Total	*P*
Age (y)						
13‐20	5 (45.5)	2 (18.2)	1 (9)	3 (27.3)	11	.13
21‐65	18 (25.4)	16 (22.5)	27 (38)	10 (14.1)	71	
Male	10 (25)	9 (22.5)	14 (35)	7 (17.5)	40	.95
Female	13(31)	9 (21)	14 (34)	6 (14)	42	
Study site						
WRNMMC	0 (0.0)	0 (0.0)	1 (100)	0 (0.0)	1	.30
SAMMC	5 (62.5)	1 (12.5)	2 (25)	0 (0.0)	8	
NMCSD	12 (27.3)	12 (27.3)	14 (31.8)	6 (13.6)	44	
NMCP	5 (22.7)	5 (22.7)	6 (27.3)	6 (27.3)	22	
MAMC	1 (14.3)	0 (0.0)	5 (71.4)	1 (14.3)	7	
Ethnicity						
White	13 (26.5)	12 (24.5)	16 (32.7)	8 (16.3)	49	.89
Black	7 (35)	4 (20)	7 (35)	2 (10)	20	
Asian	0 (0.0)	1 (25)	2 (50)	1 (25)	4	
Other	3 (33.3)	1 (11.1)	3 (33.3)	2 (22.2)	9	
Current smoker						
Yes	0 (0.0)	3 (25)	5 (41.7)	4 (33.3)	12	.05
Former	5 (45.5)	3 (27.3)	1 (9)	2 (18.2)	11	
Never	18 (31)	12 (20.7)	21 (36.2)	7 (12.1)	58	
Missing	0	0	1	0	1	
Season						
2010‐2011	2 (18.2)	2 (18.2)	4 (36.3)	3 (27.3)	11	.05
2011‐2012	11 (39.3)	8 (28.6)	5 (17.8)	4 (14.3)	28	
2012‐2013	2 (9.5)	2 (9.5)	12 (57.2)	5 (23.8)	21	
2013‐2014	8 (36.4)	6 (27.3)	7 (31.8)	1 (4.5)	22	
Military status						
Active duty	19 (27.5)	14 (20.3)	26 (37.7)	10 (14.5)	69	.41
Dependent	4 (33.3)	3 (25)	2 (16.7)	3 (25)	12	
Retired	0 (0.0)	1 (100)	0 (0.0)	0 (0.0)	1	

WRNMMC, Walter Reed National Military Medical Center; SAMMC, San Antonio Military Medical Center; NMCSD, Naval Medical Center San Diego; NMCP, Naval Medical Center Portsmouth; MAMC, Madigan Army Medical Center.

The distribution of HCoV species was as follows: 28 (34%) HCoV‐OC43, 23 (28%) HCoV‐229E, 18 (22%) HCoV‐NL63, and 13 (16%) HCoV‐HKU1. Among patients 13‐20 years of age, the most common species was HCoV‐229E (46%), whereas the most common species among adults was HCoV‐OC43 (38%). There were no differences between species with respect to sex, geographic location, race/ethnicity, smoking status, season of enrollment, or military status of cases (Table 1).

There was season‐to‐season variability in the leading causes of HCoV‐associated illness. In 2010‐2011 and 2012‐2013, HCoV‐OC43 was the most common strain identified, accounting for 36.3% and 57.2% of infections, respectively. In 2011‐2012 and 2013‐2014, HCoV‐229E was the most common strain identified, accounting for 39.3% and 36.4% of infections, respectively.

**Figure 1:**
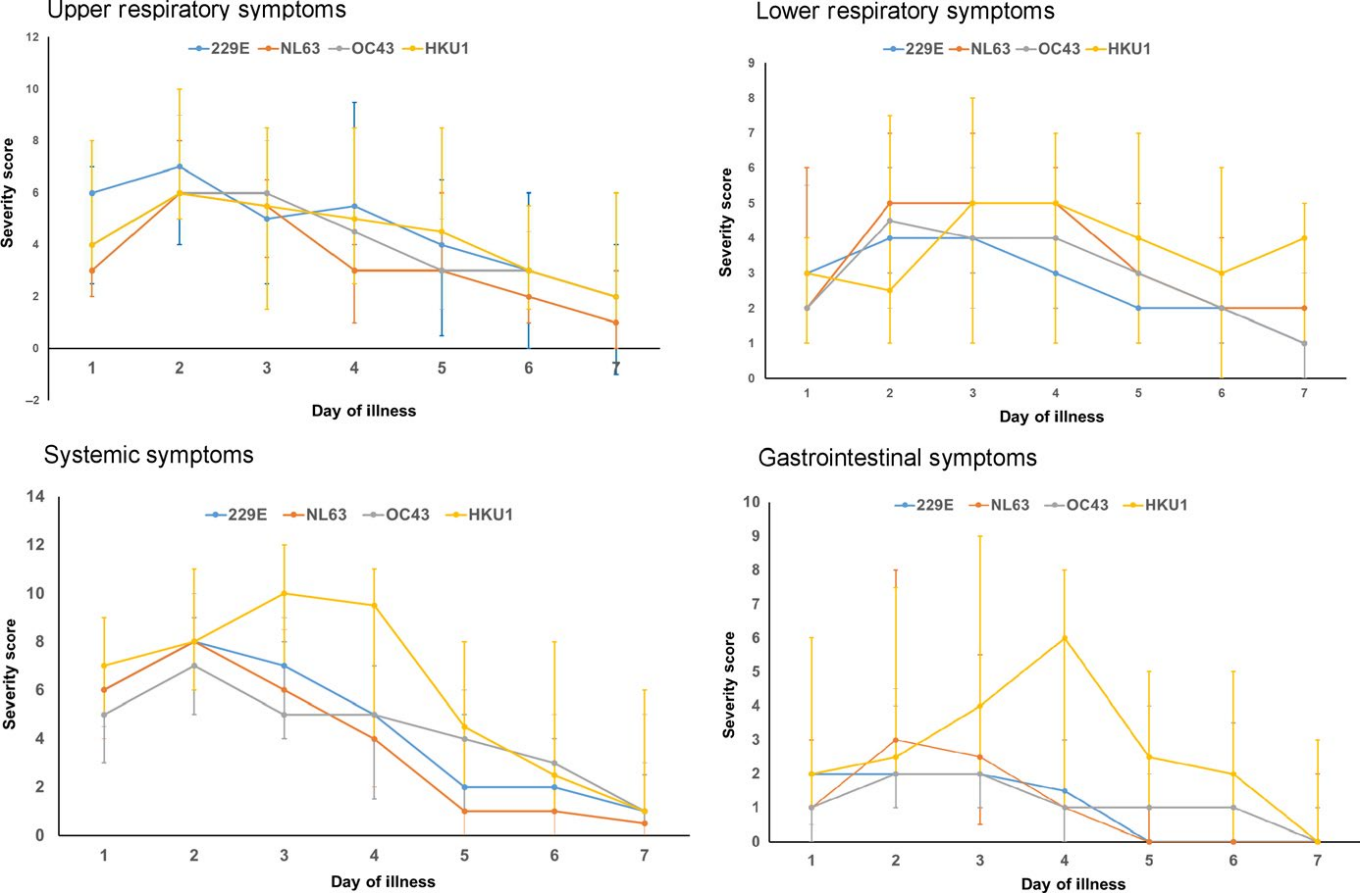
Median composite severity score by symptom category, study day, and human coronavirus species

Median daily composite symptom severity scores over the first 7 days of illness, stratified by HCoV species, are presented in the Figure 1. For all HCoV species and all symptom categories, scores peaked between study days 2‐4, and most (76%) subjects reported a persistence of symptoms through 7 days of illness. There were no differences between HCoV species with respect to the severity of upper respiratory, lower respiratory, or systemic symptoms over time. On study day 4, the median composite severity score for gastrointestinal symptoms trended higher among those with HKU1 infection as compared to the other 3 species, although the differences were not statistically significant (*P* = .05). Otherwise, the prevalence and severity of symptoms did not differ between species.

## 4 DISCUSSION

The findings of our study, conducted over a 5‐year period at five geographically dispersed sites in the USA, demonstrate that human coronavirus (HCoV) is an important cause of influenza‐like illness (ILI) among otherwise healthy adolescents and adults. Twelve percent of cases of ILI in our population were attributable to HCoV infection. HCoV‐attributable proportions in other adult populations have ranged from 4% to 22%.^8^, ^9^, ^10^, ^11^, ^14^ Additionally, we found HCoV‐OC43 to be the most common species among adults, as has been reported elsewhere.^8^, ^9^, ^11^, ^12^, ^14^

HCoV‐OC43 and HCoV‐229E were the most common strains in alternate seasons, reflecting a season‐to‐season variability of HCoV strain circulation that has been reported in other multiyear studies.^4^, ^12^ The reasons for these seasonal variations are unknown, but may reflect year‐to‐year differences in the composition of our study population with respect to age group and geographic location, in addition to other epidemiologic factors. Small sample sizes precluded an analysis of factors associated with HCoV seasonal variation.

For nearly all categories and timepoints, our longitudinal assessment of symptom presence/severity did not reveal any significant differences between the four HCoV species. There was one exception: the reported severity of gastrointestinal symptoms was higher among individuals with HCoV‐HKU1 infection, although this difference did not reach statistical significance. In a case study of adults with HCoV‐HKU1 infection, 38% reported the presence of upper or lower gastrointestinal symptoms.^8^ The mechanisms by which this particular species elicits these symptoms are not known.

The strengths of this study of HCoV in otherwise healthy adolescents and adults include its multisite and multiyear design, the use of a multiplex diagnostic panel, the prospective collection of symptom data, and the use of a symptom severity scale similar to what has been employed previously.^15^ One important limitation of this study was our selective recruitment of individuals who had presented to a healthcare facility for care of an ILI. Therefore, our cases are not representative of HCoV infection in the community, where individuals with mild, self‐limiting illness due to HCoV opt not to seek medical care for the management of their ILI.

In summary, we have shown that HCoV is a significant cause of ILI among otherwise healthy adolescents and adults presenting for medical evaluation. Although there were differences in species distribution by age group, we did not detect any differences between species with respect to the clinical spectrum of disease.

## DISCLAIMER

The contents of this publication are the sole responsibility of the author(s) and do not necessarily reflect the views, opinions, or policies of Uniformed Services University of the Health Sciences (USUHS), the Department of Defense (DoD), the Departments of the Army, Navy, or Air Force, or the Henry M. Jackson Foundation for the Advancement of Military Medicine. Mention of trade names, commercial products, or organizations does not imply endorsement by the U.S. Government. The investigators have adhered to the policies for protection of human subjects as prescribed in 45CRF46.
